# Is the NOD mouse a good model for type 1 diabetes?

**DOI:** 10.1007/s00125-025-06579-0

**Published:** 2025-11-08

**Authors:** F. Susan Wong, James A. Pearson, Li Wen

**Affiliations:** 1 Diabetes Research Group, Division of Infection and Immunity, Systems Immunity University Research Institute, Cardiff University School of Medicine, Cardiff University, Cardiff, UK; 2 Section of Endocrinology, Internal Medicine, School of Medicine, Yale University, New Haven, CT USA

**Keywords:** Immunotherapy, NOD mouse, Pathogenesis, Review, Type 1 diabetes

## Abstract

The NOD mouse is the best-known, although by no means the only, murine model of type 1 diabetes. In this review, we provide a historical perspective, particularly highlighting areas of progress in understanding aspects of aetiology and immune pathogenesis, and utility in helping to shape the immunotherapeutic landscape. We introduce points of interest where the NOD mouse, a much-studied model, has signposted discovery and knowledge. We discuss genetics, pancreatic islet beta cell stress, innate and adaptive immunity and autoantigens, and also focus on immunotherapeutic agents that have been tested in NOD mice and in humans. Some therapies, particularly those that are non-antigen-specific, have been more effectively signposted, while others, which include antigen-specific therapies, have not. There is an inevitable divergence between mice and humans that illustrates the need to use models appropriately. We suggest how to make use of this and other models effectively in order to maximise information and knowledge, and suggest not dismissing this important resource because of inappropriate comparisons or unrealistic expectations.

**Graphical Abstract:**

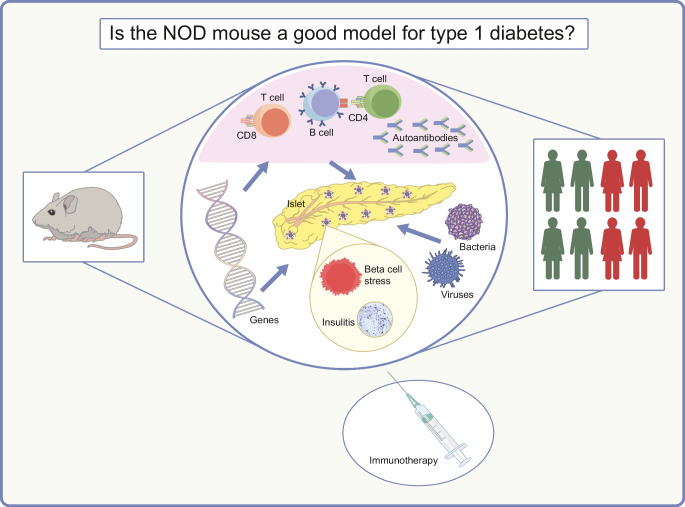

**Supplementary Information:**

The online version contains a slideset of the figures for download available at 10.1007/s00125-025-06579-0.

## Introduction

Type 1 diabetes is an autoimmune condition, clearly different from type 2 diabetes. Until the 1970s, it was known that people living with type 1 diabetes had much greater insulin deficiency, although the basis for this was not clear, despite pancreatic sections showing immune cell infiltrates [1]. Genetic association studies demonstrated that particular HLA class I complexes, A8 and B15 (earlier designated W15), were associated with the development of type 1 diabetes [2, 3]. A very large body of work followed that identified the HLA region (the human designation of the MHC) as containing the most important genetic susceptibility genes for the development of type 1 diabetes, particularly HLA class II genes. However, this is a polygenic condition, and more than 70 gene regions have been reported to contribute to susceptibility [4], although most of these have a relatively small influence on type 1 diabetes development compared with the HLA. Of note, many of the contributory susceptibility regions encode proteins involved in immune function [4]. In addition, more recent studies have highlighted genes involved in other biological functions. These include the following: (1) beta cell function and fragility (including *PTPN2*), affecting beta cell responses to cytokines; (2) *GLIS3,* regulating beta cell development; (3) *CLEC16A*, linked to beta cell function and survival [5]; (4) *BACH2*, expressed in islets where it regulates apoptosis in pancreatic beta cells and in the immune system, and expression is influenced by cytokines [6]; and (5) exocrine function (cathepsin H [CTSH], a lysosomal cysteine protease) [7].

The HLA is central to T cell function, as antigenic peptides are recognised together with specific HLA molecules by T cells (CD8^+^ cytotoxic T cells recognising peptide antigens, 8–10 amino acids in length, together with HLA class I and CD4^+^ helper T cells recognising peptide antigens of longer length, together with HLA class II). Thus, the HLA, as a major genetic susceptibility region, links to the importance of T cells in the pathogenesis of type 1 diabetes.

The major site of immune action in type 1 diabetes is in the pancreatic islets of Langerhans, which are made up of glucagon-producing alpha cells, insulin-producing beta cells, somatostatin-producing delta cells, pancreatic polypeptide-producing gamma cells and ghrelin-producing epsilon cells. However, it is the beta cells that are specifically targeted by the autoimmune response. From the 1980s, enormous strides were made to advance T cell biology including knowledge of T cell development in the thymus, T cell differentiation into different subsets, how each T cell subset functions and their roles in immune responses. T cells are major players in direct beta cell damage, although other immune cells are also indispensable, and together with soluble mediators form an inter-dependent network.

There were other important developments in understanding that human type 1 diabetes had an immune component. Autoantibodies to insulin [8], the 64K protein which was identified as GAD [9], insulinoma antigen 2 (I-A2) [10], ZnT8 [11] and more recently tetraspanin 7 [12], are now known to be present before clinical onset of the condition. High levels of two or more of the autoantibodies predict clinical type 1 diabetes onset to a large degree [13].

The NOD mouse was developed in Japan during the 1970s [14, 15] and became available for investigators in the USA in the 1980s, with the establishment of NOD mouse colonies at UCLA, Harvard University and the Jackson Laboratory in the USA, and also in Australia [15, 16]. The NOD mouse originated from the Cataract Shionogi (CTS) strain to study cataracts, in the Shionogi laboratories in Japan [15]. Researchers noticed the insulitis lesions, defined as inflammatory cells infiltrating into the pancreatic islets, which indicated that this was a model in which immune cells were likely to play an important role in damage to islet beta cells. Along with this, the mice developed hyperglycaemia. At the time, the NOD mouse was not the only model for the study of type 1 diabetes autoimmunity, as the BioBreeding (BB) rat had also been discovered [17]. However, BB rats have been less well studied as there are fewer reagents available for study and rats are more difficult to modify genetically. Moreover, the BB rat has marked lymphopenia. Although BB rats have a place as an important alternative animal model (reviewed in [18]), they will not be discussed further here.

Concurrent with these discoveries relating to NOD mice, there were important developments in our understanding of human type 1 diabetes. As mentioned, certain HLA alleles are the most important risk factor for human type 1 diabetes and the discovery that the NOD MHC class II *I-A*^*g7*^ (also known as *I-a*^*g7*^) was very similar to the high risk HLA allele *DQ8* in human type 1 diabetes [19] enhanced the importance of the NOD mouse as a good murine model for type 1 diabetes studies.

**Fig. 1:**
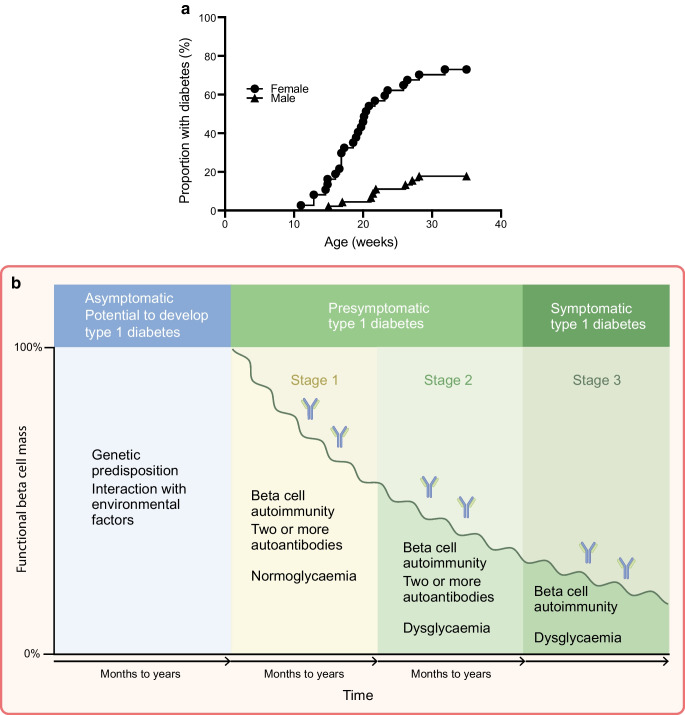
(**a**) Incidence of autoimmune diabetes in NOD mice. Spontaneous diabetes in the NOD mouse colony at Cardiff University over 2 periods of 35 weeks is shown for female mice (circles, *n*=37) and male mice (triangles, *n*=45). (**b**) Current staging of type 1 diabetes in humans based on autoantibodies and glucose abnormalities (adapted from [104] under the terms of a Creative Commons CC BY licence). This figure is available as part of a downloadable slideset

The NOD mouse develops spontaneous autoimmune diabetes. Female mice have a high incidence of between 60% and 90%, starting from age 12 weeks onwards; male mice have a lower incidence, ranging from 10% to <50%, and develop diabetes at a later age (Fig. 1a). The time course of disease development takes place over weeks, compared with the human time course that may take weeks, months or years (Fig. 1b). Immune cells infiltrate into islets, including T cells, B cells and antigen-presenting cells (APCs) such as dendritic cells (DCs) and macrophages. Although autoantibodies in NOD mice are a less prominent feature than in humans with type 1 diabetes, the mice develop autoantibodies to insulin before disease onset, with the suggestion that early production of autoantibodies may predict earlier onset of diabetes [20].

In addition to autoimmune diabetes, NOD mice have age-related hearing loss, although this is not unique to NOD mice [21], as well as autoimmunity affecting other organs. Thyroiditis is commonly found, with infiltration occurring earlier than insulitis [22]. Sialitis (inflammation in the salivary glands) is also a pathological feature [23] and NOD mice have been used as a model of Sjögren’s syndrome [24]. Thus, as in humans, the occurrence of other autoimmunity is clearly found in the NOD mouse.

The development of diabetes in NOD mice is highly dependent on the environment, and these mice require housing in specific pathogen-free conditions, where known pathogenic micro-organisms are not present. Some (although not all) pathogenic viruses and bacteria, as well as relatively ‘harmless’ infections or infestations (e.g. pinworm infection) common in conventional mouse housing, can considerably reduce diabetes incidence.

Although NOD mice without any genetic modifications have been studied for spontaneous development of type 1 diabetes, highly useful transgenic NOD mice can be generated to add particular immunological traits, or genes may be knocked out to provide gene deficiencies. More advanced and sophisticated mice may have knockin mutations in which specific genes are replaced (e.g. human HLA class I can be introduced in this way, further discussed below). Moreover, tissue-specific transgenes or knockouts have gene deletions or insertions in specific tissue types or cells; in timed gene knockouts drugs such as tetracycline are used to turn genes on or off. Furthermore, scientists can tag particular genes (e.g. with fluorescence markers) and carry out lineage tracing in vivo.

With this increasing sophistication, it is however important to note that NOD mice (and indeed many mouse models) are not simply tools that can be picked off a shelf. They have very specific requirements and, at all times, it is important for this mouse as a model of autoimmunity in general and type 1 diabetes in particular that the disease develops spontaneously, otherwise the important requirement of being a disease model is not fulfilled. Research into protection from diabetes cannot be carried out in a model that does not develop diabetes because of factors that include environmental interference.

In this review, discussion is centred on how the NOD mouse has been a ‘signpost’ towards areas of interest in, and importance for, human type 1 diabetes. This means that a discovery made in the NOD mouse may also be important to consider in human disease. It is vital not to equate ‘this is what happens in the mouse’ to ‘therefore it is so in the human’, as this will lead to disappointment and, potentially, rejection of a model that has important resemblance to, but is not a replica of, a human. The discoveries in NOD mice allow for looking in certain directions and designing experimental conditions that may open new avenues of thinking that may not have been hitherto considered in humans. Finally, NOD mice can be useful for investigating the effects of therapeutic agents with defined conditions of testing and consideration of appropriate comparison, as further discussed below.

## Pathogenesis

### Genetics

HLA class II molecules that are strongly linked to susceptibility to type 1 diabetes, particularly *HLA-DQ8* [25] (DQA1*0301-DQB1*0302), express a non-aspartate residue at position 57 of the β-chain. The discovery that the single MHC class II chain of the NOD mouse I-A^g7^ also had a non-aspartate residue, whereas other mouse MHC class II molecules expressed an aspartate residue at this position [26], gave strong support for the use of the NOD mouse as a model for human type 1 diabetes. A number of structural and functional studies showed that many peptides, including altered peptides, could bind to these MHC class II molecules in both humans and mice [19]. In addition, T cells recognise a number of possible ligands that bind with different affinities and are also present in diabetes [27]; post-translational modifications of autoantigenic peptides are also major T cell targets in type 1 diabetes [28–30]. This is important, as the impact on T cell antigen recognition and ability to evade tolerance mechanisms can be significant, as discussed later.

### Histology

**Fig. 2:**
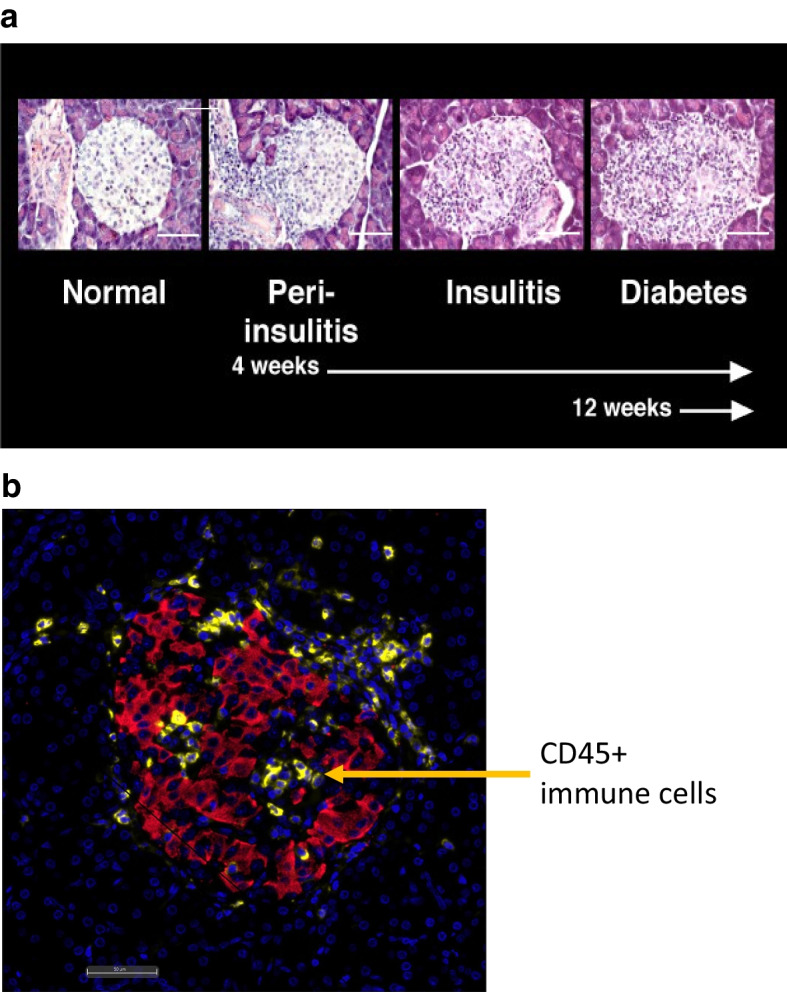
(**a**) Natural history of insulitis in NOD mice. H&E staining of histological sections showing stages of development of insulitis in NOD mice. When mice are weaned at 3 weeks of age, islets are normal. This is followed by insulitis starting from 4 weeks of age, when a mononuclear cell infiltrate begins to surround the islet (peri-insulitis), with increasing penetration into the islet (insulitis). From 12 weeks of age onwards, there is increasing islet infiltration and diabetes occurs. Scale bar, 100 μm. Image from the late Irene Visintin, Yale University. (**b**) Insulitis in human type 1 diabetes, demonstrated by immunofluorescence staining of an islet (red, insulin; yellow CD45) from an adult donor recently diagnosed with type 1 diabetes. Scale bar, 50 μm. Image courtesy of Pia Leete, Exeter University. This figure is available as part of a downloadable slideset

Histological studies of the pancreas in individuals who had developed clinical type 1 diabetes demonstrated marked upregulation of HLA class I in the pancreatic islets [31, 32], with the islets infiltrated mainly by CD8^+^ T cells [33–36]. HLA class I upregulation is a sign of inflammation in stressed cells, one cause of which may be production of IFNs, often induced by viral infection [32] (but also by endogenous dsRNA, bacteria and mycoplasma). It is associated with upregulation of signal transducer and activator of transcription 1 (STAT1), a transcription factor involved in mediating antiviral responses to IFNs [32]. This MHC class I upregulation was clearly seen in the NOD mouse, and occurs very early, even before much cellular infiltration occurs [37]. The NOD mouse has visible progression of considerable immune cell infiltration in the natural history (Fig. 2a), whereas immune cell infiltration had initially been shown to be relatively low in deceased individuals who had type 1 diabetes, based mainly on the earlier available pancreatic samples. In past years, the available pancreatic samples were mostly from individuals who had died years after diagnosis. However, more recent study shows that the level of immune infiltration in the pancreas in young individuals, diagnosed below the age of 7 years, may be similar to that seen in the NOD mouse (Fig. 2b) [36]. Understanding these similarities and differences is of paramount importance.

### Immunology

Autoimmune diabetes can be adoptively transferred by spleen cells containing both CD4^+^ and CD8^+^ T cells [38] from diabetic NOD mice into immunodeficient NOD mice (such as irradiated NOD mice or NOD.scid or NOD.RAG knockout mice). T cells can be isolated from the infiltrate into the pancreatic islets and cloned in in vitro culture. The earliest of these isolated T cells was a CD4^+^ T cell clone named BDC2.5 [39], the first of a series of BDC clones [40] contributing to the study of pathogenic CD4^+^ T cells in diabetes in the NOD mouse. After years of research, the elusive autoantigenic target of BDC2.5 was identified with the discovery that the autoantigen was not a single GAD or chromogranin A peptide alone, as had been previously suggested, but a hybrid peptide of insulin and chromogranin A [28]. This paved the way to understanding that hybrid peptides of insulin, together with another beta cell protein, could generate many potentially antigenic peptides within the beta cells. Study of hybrid peptides in humans [41, 42], as well as exploration of the mechanism of the generation of hybrid peptides in the NOD mouse [29, 43], followed. The T cells that respond to these hybrid peptides would not have encountered these antigens in the thymus and therefore central tolerance would not be induced.

Insulin and its larger precursor proinsulin were identified as targets for autoantibodies decades ago [20, 44] and have been much studied as targets for CD4^+^ T cells. Work in the NOD mouse suggested that insulin is a prime antigen and that the peptide B chain amino acids 9–23 region is a major epitope [45]. This region of the insulin B chain can be processed and presented to different sets of T cells [46], and the B chain is also an important part of hybrid insulin peptides, mentioned above [47]. Investigation into hybrid insulin peptides in humans have uncovered other peptides of the proinsulin molecule that form part of the hybrid insulin peptides that stimulate human CD4^+^ T cells [48, 49].

Although the upregulation of HLA class I was most prominent in human islets, relatively little attention was initially given to MHC class I-reactive CD8^+^ T cells, compared with the CD4^+^ T cells. β-2 microglobulin-deficient NOD mice, which expressed highly reduced levels of MHC class I, and consequently few CD8^+^ T cells, did not develop autoimmune diabetes [50, 51]. This finding coincided with the cloning of a CD8^+^ T cell (NY8.3) from the islets of a NOD mouse that was found to be highly pathogenic [52], followed by cloning of a diabetogenic CD8^+^ T cell (G9C8) from the islets of young NOD mice long before the onset of clinical diabetes [53]. The autoantigens recognised by the cloned CD8^+^ T cells were identified as a peptide of insulin B chain amino acids 15–23 [54] for the G9C8 clone from the young NOD mouse and as islet-specific glucose 6-phosphatase related protein (IGRP) [55] for the NY8.3 clone from the diabetic mice. Subsequently, both insulin [56] and IGRP [57] were identified as targets for CD8^+^ T cells in humans and, thus, the demonstration of highly pathogenic CD8^+^ T cells in NOD mice gave a direction to the examination of these autoantigens in humans.

Transgenic NOD mice have also been used to express human HLA class I transgenes [58, 59], among others, the common HLA A2 that has been associated with type 1 diabetes, and HLA A3 and B7 [60]. These have facilitated the discovery of further autoantigenic epitopes, including different peptides of insulin and IGRP in humans [59, 60]. The spotlight on post-translational modifications of autoantigens in the form of hybrid insulin peptides also applies to autoantigens for CD8^+^ T cells in humans. Peptides of islet beta cell granule proteins are autoantigenic for CD8^+^ T cells in humans, presented by *HLA-A3*; in the NOD mouse these autoantigenic peptides would be presented by MHC class I *H-2K*^*d*^* (*also known as *H2-K1*) [61]. Moreover, CD8^+^ T cells reactive to these beta cell granule protein peptides in the NOD mouse were found in the islets and caused diabetes upon adoptive transfer into immunodeficient NOD.scid mice [61]. Details of these peptides and many others may be found in a recent comprehensive resource [62].

### Beta cell stress

Beta cells are highly active metabolic cells and are subject to stress, with clear parallels in the NOD mouse and humans. Prediabetic NOD mice display evidence of endoplasmic reticulum stress (ER) in the beta cells, demonstrated by alterations in the ER structure in isolated islets, suggested to be related to activation of NF-κB [63]. Islet cells from NOD mice subjected to chemical ER stresses express peptides with calcium-induced post-translational modifications and these islets become more antigenic for diabetogenic BDC2.5 CD4^+^ T cells [64]. In human type 1 diabetes, ER stress in beta cells leads to generation of antigenic epitopes due to enzymatically induced protein modifications. CD4^+^ T cells reactive to these modified proteins have been found in peripheral blood and in the draining pancreatic lymph nodes of post-mortem pancreatic donors who had type 1 diabetes [65]. Beta cell ER stress also activates inflammatory pathways, with increased expression of proinflammatory genes and MHC molecules in the mouse and HLA in humans. These stresses cause metabolic abnormalities, impairing insulin production and secretion, resulting in hyperglycaemia, which is toxic to the beta cells. ER stress also induces apoptosis. All of these contributory factors may be found in the NOD mouse and are mirrored in human type 1 diabetes (recently reviewed in [66]). These stresses, and the interplay with the immune system, have suggested potential therapeutic strategies that will be further discussed later in this review.

### Environment

The environment affects the development of diabetes in the NOD mouse [67] and a variety of infectious agents can influence diabetes in this model. Viruses were investigated as causative agents for development of diabetes. Molecular mimicry between the enterovirus coxsackievirus P2-C (Cox P2-C) protein and GAD could contribute to damage to the pancreatic beta cells [68]. Coxsackie B4 virus causes pancreatitis and exocrine damage, and accelerates ongoing islet beta cell damage in NOD mice [69]. Viral infection induces inflammation and diabetes can occur as a result of virus-induced inflammation and bystander activation of autoreactive T cells [70]. However, these observations suggest that the virus is not causative in the NOD mouse but could contribute to acceleration of immune reactivity. In human type 1 diabetes, advances in histological techniques and increased availability of post-mortem pancreatic samples from donors who had lived with type 1 diabetes have shown indices of low-grade chronic enterovirus infection. These include the presence of enteroviral capsid protein VP1, found in pancreas samples from autoantibody-positive individuals, together with HLA class I hyperexpression, before clinical onset of type 1 diabetes [71, 72].

Conversely, viral infections [73, 74], bacterial infections [75], parasitic infections (with *Schistosoma mansoni* [76]) and infections with pinworms [77, 78] have been reported to reduce diabetes in NOD mice. The various infections stimulated regulatory pathways that regulated inflammation, reducing development of diabetes. However, it is clear that only some agents have these effects, suggesting possible therapeutic strategies based on infection or infectious agents.

Other advances occurred in understanding the interplay of the innate immune system (the immediate immune responses as the first line of immune defence) and the internal environment [79]. Commensal bacteria shape the immune system by engaging with innate immune receptors that include, among others, the Toll-like receptors (TLRs), signalling through the myeloid differentiation primary response 88 (MyD88) pathway, in dendritic cells and macrophages [80]. An increase in the development of clinical type 1 diabetes was observed in children born by Caesarean section compared with vaginal delivery (meta-analysis of earlier studies [81]), suggesting an association with commensal bacteria. Gut bacteria were highlighted as the innate immune system link with development of autoimmune diabetes in the NOD mouse [82]. Alterations in sensing the intestinal microbes via innate immune interactions are a critical non-genetically encoded factor modifying type 1 diabetes susceptibility in the NOD mouse. Manipulation of gut environment in NOD mice, by long-term treatment with antibiotics prenatally or at an early age, to alter the commensal composition, led to changes in the incidence of diabetes [83, 84]. Note, however, that these observations relating to the prenatal environment and early neonatal environment in which the immune system develops neither suggest that nor provide any evidence for taking antibiotics to treat infections in humans affects type 1 diabetes development.

In the Finnish DIABIMMUNE study including infants from Russia, Finland and Estonia, studied from birth until 3 years of age, many more children from Finland and Estonia developed type 1 diabetes compared with children from Russia, in spite of similar genetic background [85]. The endotoxin lipopolysaccharide (LPS) from *Escherichia coli* stimulated endotoxin tolerance and reduced innate immune activity, and was found more in the gut microbiota of infants from Russia. However, this tolerance was inhibited by LPS from *Bacteroides dorei*, with more *B. dorei* found in the gut microbiota of infants from Finland and Estonia [85]. Interestingly, i.p. injection of LPS from *E. coli* reduced diabetes in NOD mice, whereas the LPS from *B. dorei* did not [85].

Many autoimmune diseases have a female preponderance, although more males develop type 1 diabetes in humans [86]. In NOD mice, more female mice develop diabetes and earlier, and there is a clear hormonal influence. Gut bacteria also influence this increase in the spontaneous diabetes in female NOD mice [87–90], a finding relevant for the female bias in other autoimmune diseases.

Manipulation of gut bacteria, using a diet that enriches the concentration of short-chain fatty acids (SCFAs), could be used therapeutically [91]. The SCFAs butyrate and propionate protect against autoimmune diabetes in NOD mice, in part by enhancing the function of T regulatory cells. In one of the largest studies from seven centres in Europe and the USA, the Environmental Determinants of Diabetes in the Young (TEDDY) study, examined stool samples from 783 children (aged 3 months to 5 years). Investigators found an increase in the genes involved in the biosynthesis of SCFAs in the healthy control children [92]. Specific alteration in the gut bacteria demonstrated therapeutic success in NOD mice, and it remains an attractive (but difficult to achieve) potential future therapeutic option in humans.

A novel therapeutic approach has used *Lactococcus lactis* bacteria as a delivery vehicle, incorporating the immunoregulatory cytokine IL-10 and proinsulin, given to NOD mice orally to induce oral tolerance. While this treatment alone provided modest, non-statistically significant protection against autoimmune diabetes, its efficacy was enhanced when combined with anti-CD3 treatment, leading to sustained reversal of hyperglycaemia [93]. This treatment been studied in a small human trial, in combination with anti-CD3 therapy. It was found to be safe and showed a potential reduction in proinsulin-specific CD8^+^ T cell frequency, suggesting promise for future clinical applications [94].

Finally, molecular mimicry has been proposed as a possible mechanism for the activation of autoreactive T cells. In NOD mice, a peptide of the gut bacterium, *Leptotrichia goodfellowii* from the phylum fusobacteria, has similarity to the IGRP peptide 206–214, which stimulates pathogenic CD8^+^ T cells. The peptide from these fusobacteria activates pathogenic IGRP-specific CD8^+^T cells in NOD mice [95], accelerating diabetes in the NOD-mouse-derived NY8.3 CD8^+^ T cell receptor transgenic mouse, which expresses a large number of CD8^+^ T cells that react to the IGRP peptide. More recently, a peptide of a human gut commensal bacterium, *Parabacteroides distasonis*, with homology to the insulin B9–23 peptide, accelerated diabetes in the NOD mouse. T cells from individuals with type 1 diabetes recognise this peptide [96] and it is possible that activation of the insulin-reactive cells could be stimulated by interaction with this bacterium. Another recent study of individuals with the HLA A*2402, which has considerable homology with MHC class I *H-2K*^*d*^ of the NOD mouse, indicated molecular mimicry with a *Klebsiella* bacterial sequence that could activate pre-proinsulin reactive CD8^+^ T cells [97]. Thus, studies of the gut commensal flora of the NOD mouse have signposted a possible mechanism for activation of autoreactive T cells in humans.

## Therapy

A mouse model that develops a disease resembling some aspects of the human condition is an obvious tool for testing potential therapies. However, it is vital to acknowledge differences in physiology and that responses to therapeutic agents may be different in a mouse compared with a human. While the course of diabetes that develops in the NOD mouse has similarities to both childhood and adult-onset autoimmune diabetes, essentially clinical diabetes occurs only in adult mice (mice are sexually mature at 6 weeks but diabetes occurs from around 12 weeks onwards). It is also very important to recognise that, in NOD mice, therapeutic agents can be tested in both the preclinical stages (after weaning in early life, from 3 weeks of age) until the development of clinical diabetes (in adult life, from the age of 12−14 weeks onward). As in humans, even with insulitis present in all NOD mice, it is difficult to reliably predict hyperglycaemia onset (median onset in female mice is 20–24 weeks of age) or whether it is very unlikely that clinical diabetes will occur (after 35 weeks of age). Thus, when considering testing therapy in NOD mice, several questions should be asked:

- Stage of disease: it is likely to be much easier to halt an autoimmune response early; once there is a ‘full-scale’ immune response, are strategies that may be useful at earlier stages likely to be much less effective, especially as there has already been loss of considerable numbers of beta cells?
- Route of administration: as in humans, oral, s.c., i.v. and nasal routes can be used in mouse models; however, in animal studies the i.p. route is often used, which is not/cannot be used in humans – is there an equivalent?
- Dose and frequency of administration: considering that adult NOD mice weigh 20–30 g and that adult humans may weigh >2000 times more, should body weight be taken into account, considering its important effects on pharmacokinetics and pharmacodynamics?
- How many mice are required to achieve sufficient power? Relative homogeneity of mouse response compared with the heterogeneity of humans may influence this, and one of many power calculation tools for clinical studies should be used
- How do we determine ‘success’?

Until recently, immunological treatments in humans were given at stage 3 type 1 diabetes (i.e. after clinical manifestation, when many islet beta cells have been damaged or destroyed). At best, this allowed the remaining beta cells to recover from glucose toxicity and prevented further loss of C-peptide, the measure of endogenous insulin production. In contrast, in the NOD mouse, we can give treatments at much earlier stages, in early insulitis, long before the onset of hyperglycaemia, or nearer to the onset of clinical disease at a time when a few mice in the colony begin to develop diabetes, and beyond. Carrying out preclinical studies in the NOD mouse also provides the opportunity to investigate the mechanism(s) that may explain any successful disease reduction.

There has been considerable criticism of the NOD mouse model when the outcomes of therapies in humans were not as expected, compared with the model, even if the conditions and/or the protocols were not comparable. For example, if a tested treatment is given to mice at 4–8 weeks of age, this is the equivalent of treating a person at the first signs of development of autoantibodies at stage 1 disease (Fig. 1b), or even earlier, whereas few treatments have been tested in humans at this stage, and this phase of the disease is much longer in humans than the equivalent period in mice. Thus, when a treatment may not have shown the anticipated benefit in a human treated at stage 3, when compared with the efficacy of early treatment in NOD mouse, this will be akin to comparing ‘apples with oranges’ and, inevitably, it will be ‘disappointing’. In addition, many treatments in the NOD mouse have been given at a young age, whereas there have been relatively few clinical trials in children, and children and adults may have different responses. Reed and Herold gave some very good comparisons of earlier studies in a perspective commentary [98], and here we highlight studies in NOD mice showing ‘successful’ human translation in Table 1, and selected examples of agents not demonstrating successful human translation (but also not necessarily successful in NOD mice either) in Table 2.

**Table 1:** Therapeutic agents modulating the immune system: translation from the NOD mouse model

Treatment	Clinical trial	NOD mouse study
Anti-CD3 (teplizumab)	Delayed reduction of C-peptide in stage 3 [105, 106] Delayed onset in stage 2 [107, 108]	Reversal of diabetes in 64–80% of diabetic NOD mice; insulitis remained [109]
Anti-CD20 (rituximab)	Delayed C-peptide loss in stage 3 for 1 year but not at 2 years [110, 111]	Diabetes onset delayed and decreased at pre-diabetes; hyperglycaemia reversed in 36% of diabetic NOD mice [112] Other anti-B cell treatments [113–115] delayed onset/reversed hyperglycaemia
CTLA4-Ig (abatacept) and ATG	CTLA4-Ig slowed C-peptide loss in new-onset diabetes at 1 and 2 years [116, 117] Did not delay progression in relatives [118]	CTLA4-Ig reduced diabetes in young prediabetic mice but did not reverse hyperglycaemia in NOD mice [119, 120] Added to ATG, hyperglycaemia was reversed [120]
ATG	Low-dose ATG preserved C-peptide in new-onset diabetes; low-dose ATG+GCSF was not effective; high-dose ATG was not effective [121]	ATG reversed hyperglycaemia in diabetic NOD mice at a low dose, as well as together with GCSF [122] ATG+CTLA4-Ig reversed hyperglycaemia in all mice treated; insulitis was also reduced [120]
Anti-IL-21	Anti-IL-21 mAb alone and the GLP-1 agonist liraglutide alone had no effect on C-peptide reduction in new-onset diabetes but together they reduced C-peptide loss [123]	Anti-IL-21 mAb reversed diabetes in 9/18 diabetic NOD mice, and in 16/18 mice when combined with the GLP-1 agonist liraglutide [124]
Anti-TNF-α (etanercept, golimumab)	Etanercept preserved C-peptide in a small study in new-onset diabetes over 24 weeks [125] Similar results were obtained with golimumab in a larger study of 84 participants aged 6–21 years [126, 127]	Anti-TNF-α given to diabetic NOD mice prevented diabetes in 4/8 4-week-old prediabetic mice [128] Hyperglycaemia was reversed in 22/24 diabetic NOD mice [129]
Baricitinib	Baricitinib given orally in stage 3 type 1 diabetes preserved C-peptide over 48 weeks [130]	JAK1/JAK2 inhibitor reversed hyperglycaemia in NOD mice but hyperglycaemia returned on drug cessation [131] JAK1 selective inhibitor reversed hyperglycaemia, with normoglycaemia maintained in half the mice on treatment cessation [132]
Imatinib	Imatinib preserved C-peptide in the first year in a study in new-onset type 1 diabetes but this was not maintained subsequently [133]	Imatinib reversed hyperglycaemia in diabetic NOD mice [134] but hyperglycaemia returned on discontinuation of treatment
Anti-IL-17 (ustekinumab)	Anti-p40 of IL12 and IL23, inhibiting IL-17 production (ustekinumab) was given to 12- to 18-year-old individuals; at 12 months, stimulated C-peptide was higher in the intervention group [135]	Anti-IL-17 reduced diabetes incidence in NOD mice when administered at the prediabetic age of 10 weeks but not after the development of hyperglycaemia; IL-25 treatment, which inhibits Th17 cells, reversed hyperglycaemia [136]

ATG, anti-thymocyte globulin; GCSF, granulocyte colony-stimulating factor; GLP-1, glucagon-like peptide 1; JAK, Janus kinase; mAb, monoclonal antibody

**Table 2:** Therapies that have not translated from NOD mouse to humans or that were negative in NOD mice and in clinical trials in humans

Treatment	Clinical trial	NOD mouse study
Oral insulin	In the randomised double-blind DPT-1 study, islet-cell-antibody-positive relatives of people with type 1 diabetes, who also had first-phase insulin response above threshold but normal glucose tolerance, were given 7.5 mg oral insulin daily; no delay in type 1 diabetes was seen in the insulin vs placebo group [137] Autoantibody-negative children, aged 2–7 years, at high risk of developing type 1 diabetes, were given dose-escalated insulin orally up to 67.5 mg in a small study of 25 children who were randomised; none developed autoantibodies or diabetes, and the treatment was deemed to be safe [138] Autoantibody-negative children aged 6 months to 3 years, at high risk of developing type 1 diabetes, were given dose-escalated insulin orally, up to 67.5 mg, and did not show significant differences in pre-defined insulin responses [139]	Oral insulin was given at three different doses to 5-week-old prediabetic female NOD mice, twice weekly for 5 weeks and then weekly for 1 year. Protection was demonstrated by delay of onset and reduction of final incidence at the highest dose of 1 mg; however, diabetes incidence in control mice was low (<50%) [140] Oral insulin peptide (B chain amino acids 10–24) fed to NOD mice either when a neonate or at 4-weeks-old protected NOD mice [141] Oral porcine insulin fed to NOD mice from 5 weeks of age protected NOD mice; human insulin with a B chain amino acid 30 mutation did not provide protection [142] A larger study using human, porcine or murine oral insulin given at 5 or 9 weeks of age, twice a week at 1 mg, did not show any protection in NOD mice [143]
Nasal insulin	Nasal insulin was given to children, >1 year old, positive for *HLA-DQB1* risk alleles, positive for ≥2 autoantibodies, in a randomised double-blind study (DIPP study); no difference in autoantibody seroconversion or diabetes was observed [144] Nasal insulin was given to adults aged 30–75 years within 1 year of diagnosis in double-blind placebo-controlled study (INIT-1 study); no difference in decline of C-peptide was observed [145]	Nasal insulin administered with different protocols, including 3 or 10 days and then weekly, to female NOD mice, starting at 4 weeks of age (onset of insulitis and therefore prediabetic), protected NOD mice from diabetes development [146]
GAD-alum	Individuals newly diagnosed with type 1 diabetes aged 3–45 years were given three s.c. injections of GAD-alum or control; no difference was seen in C-peptide decline at 1 year [147] In 334 individuals, within 3 months of diagnosis with type 1 diabetes given two or four s.c. injections of GAD-alum or placebo; no difference in stimulated C-peptide was seen at 1 year [148]	i.v. GAD or intra-thymic injection of GAD antigen to 3-week-old mice protected against diabetes in NOD mice [149, 150]
IL-1RA and anti-IL-1β antibody	A study using anti-IL-1β (canakinumab) in individuals aged 6–45 years studied for 1 year, and another using IL-1RA (anakinra) in individuals aged 18–35 years studied over 9 months, showed no difference in stimulated C-peptide [100]	IL-1RA provided no protection in NOD mice and anti-IL-1β did not reverse diabetes in diabetic mice [99] With addition of anti-CD3, which has significant protective effect alone, there was enhanced protection from spontaneous diabetes [99]

IL-1RA, IL-1 receptor antagonist

There has been much focus on therapies that have not translated from protection of NOD mice against development of spontaneous diabetes to alteration of diabetes in humans (Table 2). Antigen-specific therapy has been an area of therapy particularly difficult to translate, possibly related to use of an adjuvant combined with whole antigen or peptides, route of administration or timing of therapy, as many examples of antigen-specific therapy in NOD mice have been commenced early in the preclinical phase. Furthermore, although there are clear similarities, immunological features of NOD mouse diabetes can be closely studied, whereas the immunology prior to diagnosis of human type 1 diabetes is relatively unknown. In addition, within the last 10 years, the knowledge that many autoantigenic peptides are post-translationally modified, particularly those produced within the beta cells, may make it difficult to use linear peptides or whole antigens for tolerogenic strategies.

Other areas of non-translation have included therapy targeting the innate arm of immunity, such as anti-IL1, although it should be noted that anti-IL1 treatment alone did not have an effect in NOD mice [99] but human studies were still conducted [100]. Therapy related to use of the prominent autoantigens proinsulin and GAD are outlined in Table 2.

It should also be noted that therapeutic agents tested across different laboratories have yielded variable results in some studies. It is likely that the reasons are multiple, and include the following:

- environment relating to diet, types of housing and general mouse colony management; these may all influence gut microbiota, which in turn interacts with immune responses
- environmental and genetic interactions, which may not be similar between humans and mice
- genetic drift causing selection of some gene variants, which may influence development of diabetes
- timing of administration of therapeutic agents related to disease development

Rather than blaming the mouse, or suggesting that it is not useful, mouse and human differences in physiology as well as immunology should be taken into account. Mice used for preclinical studies are mostly inbred, with much less heterogeneity between the mice. Understanding differences in the pathology and pathogenesis of autoimmune diabetes is important and should also be considered. Appropriate validation of experimental outcomes in preclinical studies in NOD mice is recommended, and the first published study should be carefully replicated. In this regard, there are calls for harmonisation of protocols and replication, prior to development of clinical trials [101].

Beyond testing potential human therapies for diabetes prevention or treatment, NOD mice can also serve as an early warning system for detecting adverse effects or even acceleration of diabetes that may signal risks for clinical translation. Early in the investigation into the role of the programmed death 1 (PD1) molecule as a negative co-stimulator, very important for peripheral tolerance, inhibition of the PD1–programmed death ligand 1 (PDL1) pathway accelerated autoimmune diabetes in NOD mice [102]. Many years later, as checkpoint inhibitor (CPI) treatment is now used for various cancers, particularly the anti-PD1 monoclonal antibody, nivolumab, about 1% of individuals receiving this treatment develop an autoimmune form of diabetes. A majority of these individuals express diabetes HLA susceptibility alleles, including *HLA-DR4* [103], and they may develop autoantibodies. New guidelines for pre-testing individuals relating to the precipitation of autoimmune diseases by these CPIs may be required.

In using NOD mice in preclinical testing of therapy, there are also other considerations, based on NOD mouse biology, as mentioned earlier, and these suggestions are shown in Table 3.

**Table 3:** Suggested considerations for studying NOD mice related to therapeutic translation

Consideration	Action	Reason
Commensal environment	Mice should be pooled from different cages and both treated and control mice should be taken from this pool	Avoidance of cage and breeder effects [151]
Metabolic environment	Many mouse studies may not use insulin for treatment once hyperglycaemia has occurred; however, insulin should be considered. In humans, insulin would be initiated at stage 3	Glucose is toxic to beta cells. Insulin therapy will be important to assist with this, and protocols for insulin delivery in animal models have been developed [152]
Administration of therapy	Dose, frequency of administration, administration route and time of day at which therapy is given, taking account of body weight compared with humans, should be considered	Comparison with administration that is practical and feasible in humans will be important
Rigour for conduct of experiments	Researchers should be blinded at the different stages of investigation, including the following: (1) administration of the treatment given to the mice; (2) monitoring the mice for diabetes; (3) the bench experiments during and at the end of treatment; and (4) at the time of scoring for insulitis	This should be applied to any therapeutic design
Experimental design	The numbers of animals used in studies should be sufficient, and the reason for selecting these numbers stated. Beyond the use of individual studies as pilot trials, power calculations should be performed [153, 154]	Ensures data are rigorous and scientific conclusions can be properly interpreted
Observational period	When assessing potential therapeutic agents for ‘prevention’ of overt diabetes, the period of observation should continue beyond the time point at which most mice develop hyperglycaemia (usually 30 weeks)	If the observation period is not sufficiently long, there could be only a minor delay rather than an effect that is deemed to be ‘prevention’
Quality control of NOD colony	Knowledge of the microbiological status by routine testing (i.e. the presence/absence of particular bacteria, viruses or parasites in a specific-pathogen-free environment) is essential. In addition, diabetes incidence can be affected by genetic drift [155]	Ensures that interventions and studies performed are due to investigated differences and not related to infection or unaccounted-for genetic changes
Reproducibility	Testing should be carried out in more than one research centre, using agreed parameters based on pilot experiments	Allows comparison of results

## Conclusions

It has not been possible to discuss all the myriad NOD mouse studies in this review, with apologies to individuals whose work has not been included. Instead, we discuss particular areas where NOD mice have been useful. We have highlighted the fact that animal models should be used judiciously and are a signpost to give direction for new areas of investigation and not a direct ‘this is so in the mouse and therefore in humans’. Many reagents and tools are available for immunological study in mice. It is crucially important to use the information as a pointer for understanding biology and potential usefulness of therapy but not for a statement of equivalence. Understanding the conditions under which NOD mice should be maintained and that they are not simply tools to be bought ‘off the shelf’ is vital. When designing experiments, consulting investigators with expert knowledge of using NOD mice may be helpful. Equally, when using the NOD mouse as a preclinical model for therapy, more attention should be given to designing studies in a manner that will allow for critical assessment of translatability. This is important, as planning for clinical trials generally requires some benefit to be shown in preclinical models, and the NOD model has been of importance in some of these.

We conclude that this is a useful model, when carefully and judiciously used, for furthering our understanding of pathophysiology and immunological processes. NOD mice may also assist in the development of therapy, based on rational scientific principles but also acknowledging the limitations of this model. Results must be further tested, aiming in the future to advance us further towards ‘prevention’, or ‘cure’, to enhance quality of life in individuals living with type 1 diabetes.

## Supplementary Information

Below is the link to the electronic supplementary material.

Slideset of figures (PPTX 456 KB)

## Abbreviations

BB: BioBreeding
CPI: Checkpoint inhibitor
ER: Endoplasmic reticulum
IGRP: Islet-specific glucose 6-phosphatase related protein
LPS: Lipopolysaccharide
PD1: Programmed death 1
SCFA: Short-chain fatty acid
