# Supplementary material for: Is the NOD mouse a good model for type 1 diabetes?
Source: Diabetologia. 2025 Nov 8;69(1):3–19. doi: 10.1007/s00125-025-06579-0 (PMC12686005; doi:10.1007/s00125-025-06579-0)
Supplement: Supplementary file 1 — Slideset of figures (PPTX 456 KB) [file 125_2025_6579_MOESM1_ESM.pptx]

## Slide 1
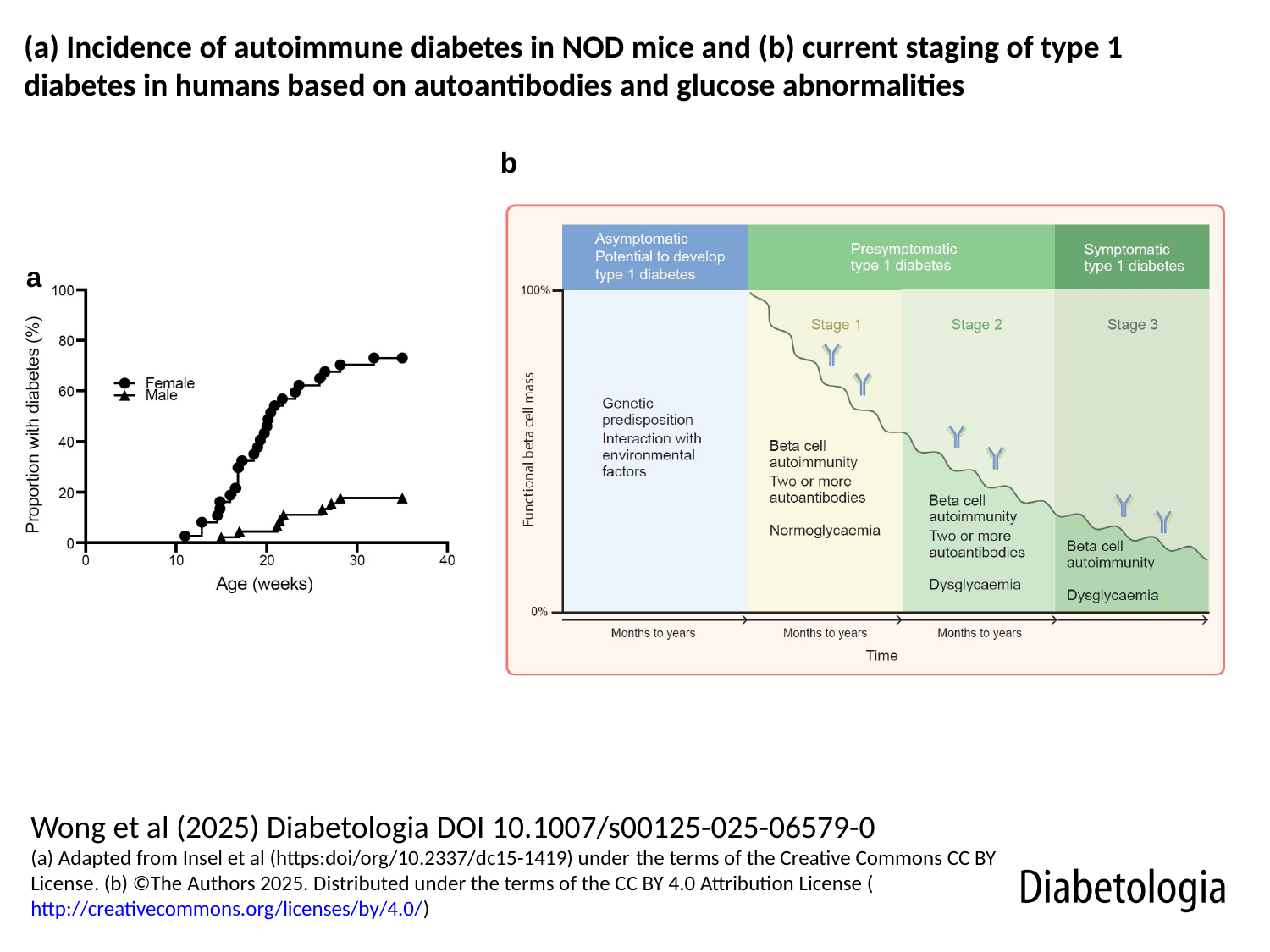

(a) Incidence of autoimmune diabetes in NOD mice and (b) current staging of type 1 diabetes in humans based on autoantibodies and glucose abnormalities
b
a
Wong et al (2025) Diabetologia DOI 10.1007/s00125-025-06579-0
(a) Adapted from Insel et al (https:doi/org/10.2337/dc15-1419) under the terms of the Creative Commons CC BY License. (b) ©The Authors 2025. Distributed under the terms of the CC BY 4.0 Attribution License (http://creativecommons.org/licenses/by/4.0/)

## Slide 2
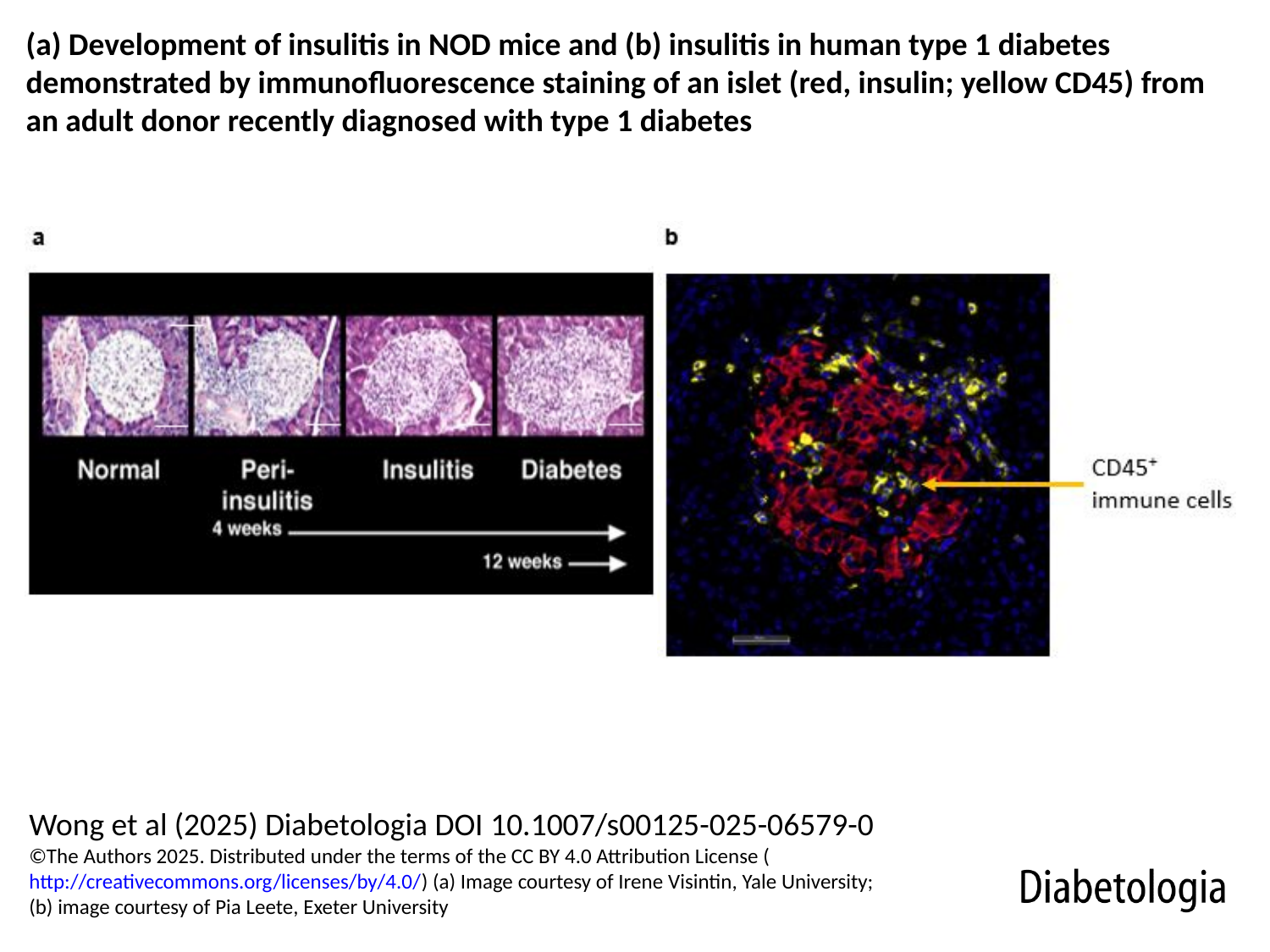

(a) Development of insulitis in NOD mice and (b) insulitis in human type 1 diabetes demonstrated by immunofluorescence staining of an islet (red, insulin; yellow CD45) from an adult donor recently diagnosed with type 1 diabetes
Wong et al (2025) Diabetologia DOI 10.1007/s00125-025-06579-0
©The Authors 2025. Distributed under the terms of the CC BY 4.0 Attribution License (http://creativecommons.org/licenses/by/4.0/) (a) Image courtesy of Irene Visintin, Yale University;
(b) image courtesy of Pia Leete, Exeter University
